# *In vitro* germ cell differentiation from embryonic stem cells of mice: induction control by BMP4 signalling

**DOI:** 10.1042/BSR20160348

**Published:** 2016-11-08

**Authors:** Zohreh Makoolati, Mansoureh Movahedin, Mehdi Forouzandeh-Moghadam

**Affiliations:** * Department of Anatomical Sciences, Faculty of Medicine, Fasa University of Medical Sciences, Fasa 74616-86688, Iran; † Department of Anatomical Sciences, Medical Sciences Faculty, Tarbiat Modares University, Tehran 14115-175, Iran; ‡ Department of Biotechnology, Medical Sciences Faculty, Tarbiat Modares University, Tehran 14115-175, Iran

**Keywords:** BMP4, co-culture, embryoid body, embryonic stem cell, germ cell

## Abstract

The present study aims to confirm and analyse germ cell-related patterns and specific gene expressions at a very early stage of cell commitment. Following the XY cytogenetic confirmation of the CCE mouse embryonic stem cells (mESCs) line, cells were cultured to form embryoid bodies (EBs). Expression pattern assessment of the mouse vasa homologue (Mvh), Stra8, α6 and β1 integrin genes in ESC and 1–3-day-old EB showed that all genes except α6 integrin were expressed in the ESC. The mean calibration of Mvh, Stra8 and α6 integrin expression significantly increased upon EB formation compared with the ESCs. During mouse embryogenesis, the signalling of bone morphogenetic protein (BMP) is essential for germ-line formation. To investigate its role in germ-line induction *in vitro*, mESCs were cultured as 1-day-old EB aggregates with BMP4 for 4 days in STO co-culture systems, in the presence and absence of 5 ng/ml BMP4. At the end of the culture period, colony assay (number and diameter) was performed and the viability percentage and proliferation rate was determined. There were no significant statistical differences in the abovementioned criteria between these two groups. Moreover, the expression of Mvh, α6 and β1 integrins, Stra8 and Piwil2 genes was evaluated in co-culture groups. The molecular results of co-culture groups showed higher–but insignificant–Piwil2 and significant α6 integrin expressions in BMP4 treated co-culture systems. These results confirmed that the EB system and the presence of BMP4 in a STO co-culture system improve the differentiation of ESCs to germ cell.

## INTRODUCTION

Pluripotent embryonic stem cells (ESCs) provide a powerful tool for studying the mechanisms of germ cell development *in vitro* [1–3]. Under appropriate culture conditions, stem cells differentiate into germ cell lineage [1–10]. Several groups have reported that ESCs differentiate into germ cells when co-cultured with the bone morphogenetic protein 4 (BMP4) producing cell [1], CF1 mouse embryonic fibroblast feeder layer [7] and SIM mouse embryo-derived thioguanine- and ouabain-resistant (STO) cell [3]. Previous studies showed that the addition of cytokines such as BMP4 also facilitate the germ cell differentiation of human [6] and mouse [11–13] ESCs. Furthermore, recent studies have reported the *in vitro* differentiation of germ cells from mouse ESCs [10], teratocarcinoma cells [8], human and mouse bone marrow stromal cells (BMSCs) [9,14]. Principally, two different methods have been reported to induce the differentiation of ESCs into germ cells, namely monolayer differentiation [15] and embryoid body (EB) formation [1–3,6,7,10,16,17]. In this line, Geijsen et al. [3] and West et al. [2] presented the system that requires the differentiation of murine ESCs into EBs and the subsequent isolation of germ cells by non-quantitative gene expression analyses at days 3–9 of EB differentiation.

The differentiation of germ cells from stem cells is accompanied by the switching of embryonic gene expression to germ cell specific gene expressions [2,3]. The goal of this study is to identify germ cell gene expression changes. Germ cell genes are composed of two sets of gene families, each including gene families that tend to appear in the process of germ cell formation. Thus, two sets of genes are predicted to respond to our culture systems: (i) germ cell-related genes and (ii) germ cell-specific genes. Mouse vasa homologue (Mvh), stimulated by retinoic acid gene-8 (Stra8), and piwi (*Drosophila*)-like 2 (Piwil2) are examples of the first group and α6 and β1 integrins belong to the second group [3,13].

In the present study, we compared the efficiency of the EB system without any induction; in induced germ cell development from ESCs at days 0–3 of EB formation and differentiation. Mvh, Stra8, α6 and β1 integrins expression profiles, as indicators of the germ cell differentiation pattern [18] were evaluated quantitatively. In addition, the effect of BMP4 in STO co-culture system on germ cell derivation was investigated using germ cell gene expression analyses.

## MATERIALS AND METHODS

### Cell line

CCE (by Dr John Draper, Stem Cell Center, Sheffield University) is a mouse embryonic stem (ES) cell line derived from 129/Sv mouse strain. The CCE cell line has been adapted to grow on gelatin-coated culture ware with the appropriate medium and does not require a primary embryonic fibroblast (PEF) feeder layer [19,20]. As ES cells are used as a model for male germ cell development during EB differentiation, the XY cytogenetics of this cell line demands prove. Thus, the marker of sex determination, SRY, was studied through the PCR technique.

To enhance the sensitivity of contaminant detection, the marker of pluripotency, Oct-4, was studied and the pluripotency of CCE mouse ESCs was confirmed with a positive Oct-4 immunocytochemistry reaction.

### Cell culture medium and reagents

Undifferentiated CCE mouse ESCs were cultured at 37°C with 5% CO_2_ and 95% humidity. The medium for cell culture included Dulbecco's modified Eagle's medium (DMEM) with high glucose, pyruvate and L-glutamine (Gibco) supplemented with 20% FBS (Gibco), 0.1 mM non-essential amino acids (Sigma), 100 unit/ml penicillin, 100 μg/ml streptomycin (Gibco), 3.7 gr/l NaHCO_3_ (Sigma), 0.1 mM β-mercaptoethanol (Sigma) and 1000 unit/ml leukaemia inhibitory factor (LIF; Sigma). The medium was renewed every day.

### Passage and maintenance of ES cells

The passage of ES cell line is conducted prior to the growth medium becoming acidic and before the cells reach confluence. Undifferentiated CCE mouse ESCs were passaged every other day. For passage, all media was aspirated from the culture vessel and dishes were rinsed with PBS. Room temperature trypsin (0.25%; Merck, Germany)/EDTA (1 mM; Sigma) was added sufficient to cover the cells. Incubation at room temperature (20°C) occurred until cells lifted off from the plate and pipetting performed for cell suspension preparation. Cells were harvested into a tube containing a solution of DMEM–15% FBS and centrifuged to pellet the cell suspension (1200 rpm=200 g for approximately 8 min). Supernatant was aspirated and the pellet re-suspended in approximately 2 ml of ES maintenance medium (DMED supplemented with 20% FBS). Pipetting was conducted until cell pellet disrupted to a single cell suspension and cell suspension divided on to a new tissue culture dish.

### Embryoid body formation

**Figure 1:**
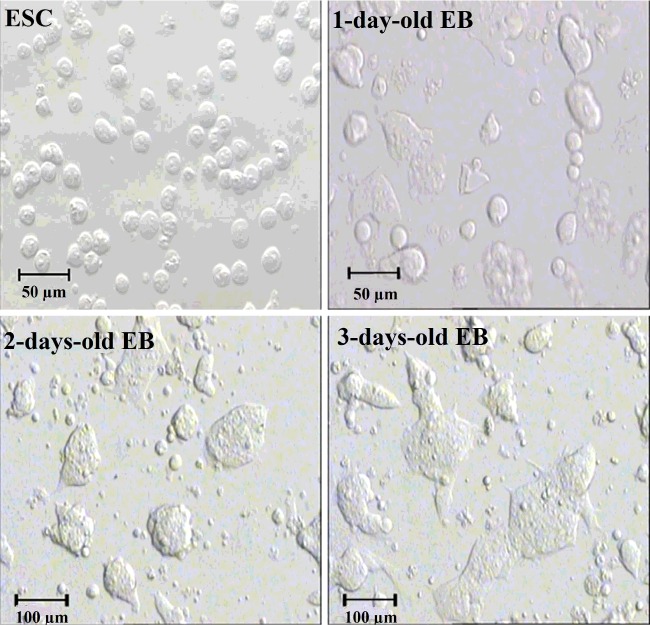
Morphology of the CCE mouse ESC and 1–3-day-old EBs. Pictures were taken with the indicated resolution at the mentioned time points

Tripsinized mouse ESCs (2×10^5^) were grown on 6-well low attachment plates in ES maintenance media for 1 day and LIF was removed. EBs formed within a few hours of suspension culture after which differentiation strategy was employed (Figure 1).

### Co-culture with feeder layer

Confluent layer of STO cells were inactivated with 10 μg/ml mitomycin C (Sigma–Aldrich) for 3 h and rinsed with PBS for three to four times. Approximately 3×10^5^ dissociated EB cells were transferred into 24-well Millipore insert co-culture plates (Nunc) on top of the mitomycin-treated STO cells and cultured in the presence and absence of BMP4 as Co-CB (Co-culture with BMP4) and Co-C (co-culture) groups respectively. Seven doses of BMP4 0.01, 0.1, 1, 5, 25, 50 and 100 ng/ml were assayed and the 5 ng/ml was chosen [21]. Culture duration was 4 days and medium renewed daily.

### Colony assay

The number and the diameter of spermatogonial cell derived colonies were assessed at the 14th day of culture in Co-C and Co-CB groups using an inverted microscope (Zeiss). Ocular grid was used to measure the diameter of colonies.

### Assessment of proliferation rate and viability percentage

The cells in the STO Co-C system both in the presence and absence of BMP4 were prepared as a single-cell suspension at the end of the culture period and stained with 0.4% Trypan blue (Sigma). Using a haemocytometer, the mean number of whole and living cells was considered as proliferation rate and viability percentage respectively.

### RNA isolation and reverse transcription

RNA was extracted from testis, CCE mouse ESC, 1–3-day-old EBs, Co-C and Co-CB groups. Total RNA was isolated using an RNX-Plus™ (Cinnagen) according to the manufacturer's suggested protocol. The concentration of extracted RNA was determined using the absorbance value of 260 nm in UV spectrophotometer (DPI-1, Kiagen) and adjusted to a concentration of 500 ng/ml. Purity of the samples was evaluated using the *A*_260_/*A*_280_ nm ratio with expected values between 1.8 and 2.0. The extracted RNAs were treated with DNase I, to remove genomic contamination, before cDNA preparation using Fermentas kit according to the manufacturer's instructions. Single-stranded cDNAs were prepared with RevertAid™ First strand cDNA synthesis kit (Fermentas) using oligo (dT) primers. The SRY gene was amplified using specified primer and β2m gene was used as an internal control (Table 1). PCR reactions were performed using 12.5 μl PCR master mix (Cinnagen), 2 μl of reverse-transcribed cDNA product and 1 μl of each primer. The reaction volume was made to 25 μl with H_2_O. The PCR reaction cycles were adjusted as following condition: 94°C for 5 min, followed by denaturation at 94°C for 20 s, annealing at 58.5°C for 30 s and extension at 72°C for 30 s for 40 cycles. The PCR products were ran on a 1.5% (w/v) agarose gel (Isolab) stained with 1 mg/ml ethidium bromide (Sigma), visualized under UV light and photographed.

**Table 1:** Primers sequences, accession numbers, expected product size and melt temperatures of germ cell, sex determinant and housekeeping genes (bp, base pair)

Accession number	Gene	Primer (forward/reverse)	Product size (bp)	Product melt temperature (°C)
NM_001145885	Mvh	5′-GCTCAAACAGGGTCTGGGAAG-3′	145	74.2
		5′-GGTTGATCAGTTCTCGAG-3′		
NM_008397	α6 integrin	5′-GAGGAATATTCCAAACTGAACTAC-3′	399	77.2
		5′-GGAATGCTGTCATCGTACCTAGAG-3′		
NM_010578	β1 integrin	5′-GTGACCCATTGCAAGGAGAAGGA-3	216	75.2
		5′-GTCATGAATTATCATTAAAAGTTTCCA-3′		
NM_009292	Stra8	5′-TCACAGCCTCAAAGTGGCAGG-3	441	77.4
		5′-GCAACAGAGTGGAGGAGGAGT-3′		
NM_021308	Piwil2	5′-GCACAGTCCACGTGGTGGAAA-3′	681	81.8
		5′-TCCATAGTCAGGACCGGAGGG-3′		
NM_011564	SRY	5′-TTG5′-CCACTCCTCTGTGACACTTTAGCCCTCCGA-3′	273	86.3
		5′-CCACTCCTCTGTGACACTTTAGCCCTCCGA-3′		
NM_009735	β2m	5′-TGACCGGCCTGTATGCTATC-3′	316	77.6
		5′-CACATGTCTCGATCCCAGTAG-3′		

### Quantitative real-time PCR

After cDNA synthesis from total RNA, optimization procedures for annealing temperatures of the primers and specific products were performed by PCRs to verify the reaction conditions. PCR reactions were performed using PCR master mix (Cinnagen) and SYBR Green in a Rotor-Gene3000 thermocycler. The primer pairs used were designed according to Toyooka et al. [1], Sutherland et al. [22] and Lee et al. [18] articles and synthesized by Cinnagen Company. β2m was used as the housekeeping gene based on that it was the most stably expressed gene in very similar experiment settings [23,24]. Real-time PCR was carried out for 40 cycles of 94°C for 20 s, 58.5°C for 30 s and 72°C for 30 s. Testis was carried out as positive control. Efficiency was determined using standard curve (logarithmic dilution series of testis cDNA) for each gene and amplification of predicted fragments was confirmed by melt-curve analysis. To determine the ratio of gene expression, we used the comparative CT (cycle threshold) method [25]. In ESC, target genes expression of Mvh, Stra8, α6 and β1 integrins was normalized to housekeeping gene. However, in 1–3-day-old EBs stages, the expression ratio (target gene/housekeeping gene) of these genes was calculated, calibrated to the ESC stage and compared between the different stages. Gene expression ratios in Co-C and Co-CB groups were calculated and calibrated to these normalized and calibrated gene expressions in one-day-old EBs. Sequences of the primers used for RT-PCR are listed in Table 1.

### Statistical analysis

Statistical analysis was carried out with SPSS 13.0. Data represent the mean of three separate experiments. The results were compared by one-way ANOVA and Tukey posttest to determine the statistical significance in the level of *P*≤0.05. Also, partial eta squared (η^2^_p_) was used as the effect size as follows: the values under 0.2, 0.2–0.5, 0.5–0.8 and higher than 0.8 were considered as weak, moderate, large and very large effect sizes respectively [26].

## RESULTS

### Expression of SRY

**Figure 2:**
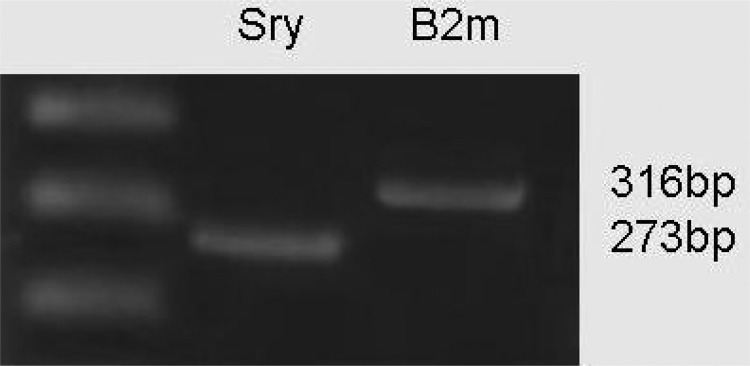
RT-PCR analysis of gene expression of Sry (273 bp) and β2m (316 bp) in CCE mouse ESC

PCR proved that Sry encoding gene was expressed by CCE mouse ES cells. This result confirmed the XY cytogenetic of this cell line (Figure 2).

### Gene expression analysis in ESC and EBs

The results obtained from this study showed that Sry, Mvh, Stra8, Piwil2, α6 and β1 integrins were expressed in testis. Expression of Mvh, Stra8, α6 and β1 integrins was investigated using quantitative real-time PCR (RT-qPCR) in the CCE mouse ES cell line and 1–3-day-old EBs.

**Figure 3:**
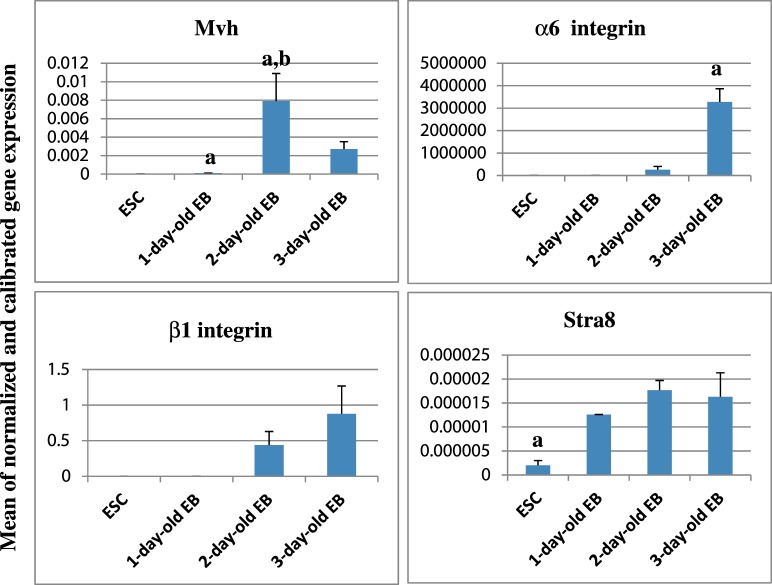
Expression profile of Mvh, Stra8, α6 and β1 integrins gene upon EB formation and differentiation RT-qPCR was done using cDNA from ESCs, 1–3-day-old EBs and testis as positive control. The mean of normalized and calibrated gene expression (*y*-axis) was shown during days of EB formation and differentiation (*x*-axis). β2m was used as normalizer. Bars represent S.D. In the Mvh graph, **a** shows significant difference with ESC and **b** indicates significant difference with 1-day-old-EB. In the α6 integrin chart, **a** shows significant difference with other groups, and in the Stra8 diagram, **a** demonstrates significant difference with ESC (*P*<0.05).

In CCE mouse ESC, expression of the target genes Mvh, Stra8 and β1 integrin was seen. The mean normalized expressions of Mvh, Stra8 and β1 integrin were 10±10^−5^, 2×10^−6^ ± 2×10^−6^ and 3×10^−5^ ± 10^−4^ respectively; but no expression of α6 integrin was observed in this cell line (Figure 3).

RT-qPCR showed that genes encoding Mvh, Stra8, α6 and β1 integrins were expressed in 1-day-old EBs. The amplified ratio of Mvh, Stra8, α6 and β1 integrin expressions relative to ES cells increased by 10.7, 6.34, 4.17 and 47.85 times respectively (Figure 3). The up-regulation of the Mvh and Stra8 was statistically significant (*P*≤0.05).

In 2-day-old EBs, quantitative PCR showed 74.58-, 1.4-, 62716.41- and 307.82-fold increases relative to 1-day-old EBs in Mvh, Stra8, α6 and β1 integrins mRNA expression respectively (Figure 3). The increases in the ratio of Stra8, α6 and β1 integrins was insignificant (*P* > 0.05), whereas Mvh gene demonstrated significant constitutive expression (*P*≤0.05). Also, the increases in the ratio of Mvh and Stra8 were significant relative to ESCs.

Gene expression analyses in 3-day-old EB displayed non-significant decrease in Mvh expression compared with the 2-day-old EBs (0.34-fold) (Figure 3). Decreased expression was also seen in Stra8 gene (0.92-fold) (Figure 3). Down-regulation of Stra8 was not significant compared with the 2-day-old EB, but this constant level of Stra8 showed significant differences with ESCs. In contrast, significant increase in α6 integrin relative to all previous stages (Figure 3) and insignificant increase in β1 integrin mRNA expression (Figure 3) was observed in this stage (12.53- and 1.99-fold increases respectively).

### Colony assay in Co-C and Co-CB groups

The number and diameters of the colonies were compared in Co-C and Co-CB groups (Table 2). Fourteen days after culture, there were no significant differences in the mean number (*P*=0.778) and diameters (*P*=0.922) of colonies among these two groups.

**Table 2:** The comparison between the mean ± S.D. of the viability percent, proliferation rate, number and diameter of colonies in the STO co-culture (Co-C) and STO co-culture with BMP4 (Co-CB) groups

Groups	Viability (%)	Proliferation rate	Number of colonies	Diameter of colonies (μm)
Co-C	47.1±1.7	5.2±0.6	142.5±5.3	198±0.8
Co-CB	49.95±3.7	2.9±0.5	176±1.5	171±0.5

### Evaluation of proliferation rate and viability percentage

The proliferation rate and viability percentage of cells in Co-C and Co-CB groups are presented in Table 2. There were no statistically significant differences in viability percentage (*P*=0.474) and proliferation rate (*P*=0.174) between the abovementioned groups.

### Gene expression analysis in Co-C and Co-CB groups

**Figure 4:**
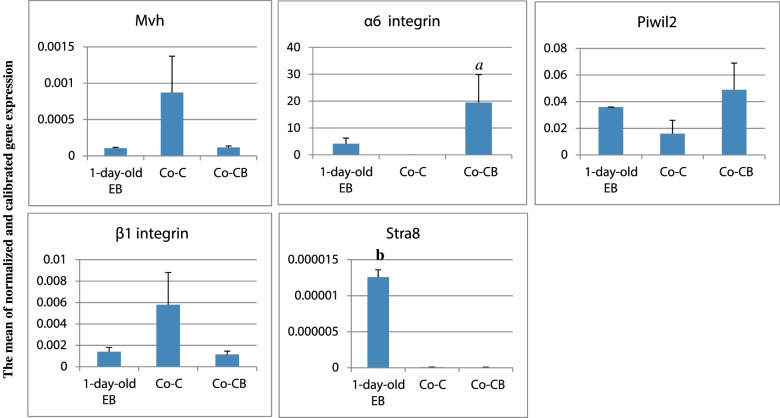
Ratio of Mvh, α6 and β1 integrins, Stra8 and Piwil2 expression relative to 1-day-old-EB in STO co-culture (Co-C) and STO co-culture with BMP4 (Co-CB) groups (mean ± S.E.M.) Shown is quantitative PCR using SYBR Green. Mean normalized expression is relative to β2m. **a** shows significant difference with 1-day-old EB and Co-C group and **b** indicates significant difference with Co-C and Co-CB groups (*P*<0.05).

Quantitatively, variations were observed in the expression of germ cell genes in Co-C and Co-CB groups. The mean normalized and calibrated expression of Mvh, α6 and β1 integrins, Stra8 and Piwil2 in the 1-day-old EB were 1.07×10^−4^ ± 1.8×10^−5^, 4.17±3.61, 1.4×10^−3^ ± 6.5×10^−4^, 1.26×10^−5^ ± 0 and 3.6×10^−2^ ± 0 respectively (Figure 4)*.*

The ratio of Mvh, α6 and β1 integrins, Stra8 and Piwil2 expression in Co-C group relative to 1-day-old EB were 8.15-, 0-, 4.18-, 0.008- and 0.45-fold, in that order. In the Co-CB group, the ratio of Mvh, α6 and β1 integrins, Stra8 and Piwil2 expression relative to 1-day-old EBs were 1.1, 4.67, 0.83, 0.0038 and 1.37, correspondingly (Figure 4)*.* Significant decrease in Stra8 expression was observed in Co-C and Co-CB groups compared with 1-day-old EBs (*P*<0.05, Figure 4). In addition, the results indicated that the ratio of α6 integrin and Piwil2 expression relative to 1-day-old EB of Co-CB group was higher than that of the Co-C group and 1-day-old EB (*P*<0.05, Figure 4). Although this increase was not statistically significant in the level of *P*≤0.05, η^2^_p_ was 0.67. This effect size quantifies the significant difference between two groups, and may therefore be said to be a true measure of the significance of the difference. Also the amplified ratio of α6 integrin expression of Co-CB group was 4.67 against the zero expression in Co-C group, significantly higher than the Co-C group and 1-day-old EBs (*P*<0.05, Figure 4).

## DISCUSSION

In this study, germ cell differentiation was demonstrated from mouse ESC upon the process of EB formation and BMP4 induction. ESCs form aggregates of cells called EBs that can spontaneously differentiate into cells of all three germ layers such as primordial and more mature germ cells [1–3]. To purify the definitive germ cell lineage in developing EBs, it is important to recognize markers differentially expressed along the germ cell differentiation [27]. Further, in order to improve culture conditions for optimal spermatogenesis, quantitative assessment of male germ cell gene expression profile is necessary [28]. Our quantitative RT-PCR analysis showed that spontaneous differentiation of ESCs into EBs caused a subsequently increase in the expression of α6 integrin concerned in germ cell development [29,30]. We also observed the increased expression of β1 integrin up to 3-day-old EB. These results were in agreement with those of previous investigations, who showed that α6 and β1 integrins were required for germ cell differentiation [18,29–31]. In this field, Ohbo et al. [27] showed that these germ cell surface markers are expressed throughout all the germ cell stages from embryo to adult and increased up to spermatogonial stem cell stage. *In vivo* germ cell differentiation has been controlled by sequential regulation of genes within the testis that is affected by cell–cell contact [32]. Shamblott et al. [28] claimed that EB provide an environment in which several early developmental processes are recapitulated, and a wide variety of lineages emerge from precursor–and more fully differentiated–cells collected randomly in these combination of cells. Taken together, increased expression of α6 and β1 integrins during EB formation and differentiation suggest the progressive germ cell lineage differentiation in EB system. Recently, some researchers showed using non-quantitative PCR that EBs derived from mouse and human ESCs express specific markers of germ cells [1,3,7,15]. Geijsen et al. [3] added retinoic acid to EBs and isolated PGC-like cells from these aggregates of cells based on RT-PCR for Piwil2, Rnf17, Rnh2, Tdrd1 and Tex14 that are germ cell-specific genes. Toyooka et al. [1] used male knockin ESCs in which LacZ or GFP was inserted adjacent to the Mvh and isolated PGCs from EBs based on Mvh expression. After transplantation of these cells into testis, fully differentiated sperm was produced. Our results showed that Mvh was expressed in undifferentiated ES cells and increased upon the process of EB differentiation up to 2-day-old EB. A constant expression of this gene persisted in 3-day-old EBs. Similarly, Toyooka et al. [1,4] showed that Mvh expression increased up to the germ stem cell stage and constant level of this gene remains until postmeiotic germ cell formation. A product of the Mvh gene is a cytoplasmic protein induced by the somatic cells of the genital ridge. Our results of Mvh expression profile confirmed those of α6 and β1 integrins, suggesting that differentiating EB acts similar to early embryo in which PGCs and more mature germ cells are formed. Our results also showed the expression of Stra8 in CCE mouse ESCs and increased expression of this gene was observed very early in EB development. A similar result was obtained by Silva et al. [33] in a study showing that Stra8 gene was expressed in undifferentiated TL-1 Sv129 mouse XY ES cells and increased with EB formation and differentiation. Mouse Stra8 express in male germ cells from E14.5 to spermatogonia. Expression of this gene is limited to the male developing gonads during mouse spermatogenesis and to the premeiotic germ cells in adult testis [34]. Generally, it seems that the same events of *in vivo* may occur during EB differentiation and some differences in the expression of genes between *in vivo* and *ex vivo* may relate to the different microenvironments of these two systems.

Additionally, quantitative PCR data in co-culture groups showed a higher ratio of germ cell-related gene expressions in the Co-CB group relative to the Co-C group (*P*<0.05), indicating that the addition of BMP4 to the culture medium promotes germ cell differentiation from mouse ESC. These results confirmed those of previous investigations, which showed that BMP4 was specifically required for germ cell differentiation [1,6,35–39]. Kee et al. [6] proved that BMPs induce germ cell differentiation from human ESC. Lawson et al. [35] reported that mutation in BMP4 genes of 7.5–9.5 days postcoitum (dpc) mouse epiblasts results in no primordial germ cell (PGC) development. It was also reported that, 72 h co-culture of 6–6.25 dpc epiblasts with BMP4 producing cells increased PGC number [36,37]. In other investigations, it was shown that the addition of BMP4 to 5–6.5 and 7.5–8.5 dpc epiblasts in the presence or absence of STO feeder layer cells caused the appearance of PGCs [38,39]. In addition, appearance of the Mvh positive cells after one day co-culture of mouse ESCs containing Mvh-reporter with BMP4 producing cells was reported by Toyooka et al. [1].

In conclusion, our results confirmed the BMP4 role for germ cell specification from mouse ESC.

## Abbreviations

BMP: bone morphogenetic protein
DMEM: Dulbecco's modified Eagle's medium
dpc: days post coitum
EB: embryoid body
ESC: embryonic stem cell
LIF: leukaemia inhibitory factor
Mvh: mouse vasa homologue
Piwil2: piwi (*Drosophila*)-like 2
RT-qPCR: quantitative real-time PCR
Stra8: stimulated by retinoic acid gene-8
